# Letter from Professor Martins

**Published:** 1840-05-02

**Authors:** 


### FOREIGN CORRESPONDENCE.

### LETTER FROM PROFESSOR MARTINS.
### No. V.

*Examination of some recent investigations on the Capillary Circulation.*

PARIS, 13th March, 1840.

To the Editors of the Medical Examiner.

THERE is, to the physician, no more interesting chapter in all physiology than that which treats of the motion and modifications of the blood in the capillary vessels. It is here that take place all the great phenomena of inflammation, and of the transformation of tissues which constitute two-thirds of the more serious diseases with which mankind are affected. Important researches have recently been made on this subject, by Messrs. Müller of Berlin, Weber of Leipsic, Huschke of Jena, and Poiseuille of Paris. Having had the good fortune to see their preparations, and to assist at some of their experiments, it has occurred to me that I might afford some interest to your readers by presenting them to their notice in this letter.

The diameter of the capillary vessels varies, according to Messrs. Müller and Weber, from 0.00025 to 0.00050 of a line. This diameter is smaller than that of the thinnest excretory ducts of the kidneys, mammæ, salivary glands, and testicles. The closest network of vessels is found in the lungs, and the choroid coat of the eyeball. While every where else, the intervals separating the vessels have a diameter superior to that of the vessels themselves, it may be asserted, that here the diameter of the intervals is equal or inferior to that of the vessels. It has been a subject of considerable discussion whether the fact of the serous membranes having received from the blood vessels the injections of Blenlaud, has placed this point beyond doubt. The experiments of Messrs. Müller and Weber have established two facts which are not of less importance. The first of these anatomists has shown that all the excretory ducts of the glands terminate in a cœcum, or cul-de-sac, and that there exists no communication between them and the capillaries, as Haller had supposed. The second has demonstrated that there is no anastomosis between the capillary vessels of the placenta and those of the uterus in the pregnant female; that the circulation of the fœtus and that of the mother are not identical, but simply in contact; and, that the passage of the blood from one to the other takes place only by imbibition. I have had the advantage of seeing the numerous preparations which prove that at the moment of the separation of the placenta there is no solution of vascular continuity, and that the hæmorrhage is not owing to the rupture of the vessels.

M. Poiseuille, in a memoir which received the prize at the Institute, has investigated the causes and phenomena of the movement of the blood in the capillary vessels. He at first examined the movement of this liquid in a part separated from the influence of the heart, either by a ligature or by removal from the body of an animal. The following are the facts discovered by him: If, by means of two weights, a portion of a capillary artery or vein be isolated in the mesentery of a frog, so that about half an inch be comprised between these two weights, the circulation is interrupted; then these vessels contract gradually upon themselves, owing to the elasticity of their parietes. Their calibre, therefore, just as that of the large arterial trunks, is owing to the pressure of the blood from within, which distends them in every direction. Now, if the femoral artery and vein of a frog be prepared for the space of half an inch, and, if by means of a strong ligature, all the parts of the thigh be enclosed, except the nerves and blood vessels, it will be seen, by means of the microscope, that the circulation continues to take place in the interdigital spaces. Let the course of the blood be then suddenly arrested in the femoral artery alone, and the globules of blood will be seen to move slowly in the capillaries, and stop at the expiration of three minutes and a half; as soon as the compression is removed, every globule starts off like an arrow. Thus, then, the globules, separated from the action of the heart by a ligature, continue to move for a certain time, from the effects of the elasticity of the parietes of the vessel, but the circulation does not go on as before. If the femoral *vein* be tied, the progress of the globules in the vessels continues some seconds, then stops, and is transformed into a sort of come-and-go movement. Finally, if the artery and vein be compressed together, there is absolute immobility. The author has thus, by experiment, verified two important facts in the history of traumatic hæmorrhages. It is, that, in capillaries cut by a sharp instrument, the blood moves in the same direction as in the arteries and veins, namely towards the amputated extremity. This is, besides, a natural consequence of the contractility of the vascular parietes, which is no longer counterbalanced by the influence of the heart. If a part be entirely separated from the body, there is, in the same manner, a flow from the orifices of the open vessels, which is due to the same cause. The author afterwards satisfied himself that the number of jerks of the globules in the capillary vessels is equal to, and isochronous with that of the pulsations of the heart. If this organ be suddenly removed from a living frog, the blood takes a retrograde movement in the arteries: that which it had in the veins continues in the same direction, but is notably accelerated, since the pressure to which the blood is subjected in the vessels from the action of the heart is superior to that of the atmosphere. If merely the ventricles of the heart be tied at its base, the same phenomena take place, though in a less marked manner. It is, therefore, to sum up, to the influence of the action of the heart that is owing the circulation of the blood, in the capillaries as well as in the arterial and venous trunks which they connect.

M. Girard, the celebrated civil engineer, had long ago said that, in tubes at rest, of small diameter, the walls of which are susceptible of being moistened by the liquid moving in them, there lay around the walls of the tube a stratum of motionless liquid, which surrounded on all sides the liquid in motion, and separated it from the walls of the tube. M. Poiseuille has established the same fact with regard to the blood vessels. If they be carefully examined by the aid of the microscope, it is seen that from the axis to the parietes of the vessel, the globules are possessed of very different velocities. In the axis the velocity is at its maximum. Close to the parietes we distinguish a very transparent space, where globules rarely show themselves. This space has a breadth about equal to the eighth or tenth of the diameter of the vessel. If we follow the course of the globules in a vessel, the diameter of which allows the passage of three, five, twelve, twenty globules abreast, the velocity of the globules in the axis of the vessel is the greatest; this velocity diminishes more and more as we approach the transparent space. In the axis and its neighbourhood, the globules have merely a forward movement; but near the parietes they have both a forward and rotatory movement, this last movement being more decided the more we approach the motionless stratum. Those which touch it, turn, so to say, upon it, and move forward much less rapidly than those of the axis of the vessel. This transparent part of the vessel, comprised between the course of the globules and their parietes, is owing merely to the presence of the serum belonging to the blood. Thus of the blood, which is incessantly in motion in all the vessels, neither the solid portion, the *globules*, nor even the liquid, or the *serum* touch the parietes. A layer of serum, by its immobility, protects them from the wear and tear which would result from direct friction against them. M. Weber distinguishes in the blood two sorts of globules; the sanguineous globules, which are elliptical, and the lymphatic globules, which are spheroidal; according to him, the latter only turns on the layer of serum. M. Poiseuille has since satisfied himself that cold retards the circulation in the capillaries, while heat retains it. He, therefore, infers that, in cold weather, other things being the same, the circulation is accomplished with more difficulty than in warm. Hence inflammations are as numerous in the latter as they are rare in the former season. Rheumatism of any part of the body unquestionably owes its existence to the arrestation of the globules consequent on the cold produced by the evaporation of the perspiration, when this part has been exposed to a current of air. But these considerations will be discussed in a work which the author is preparing on Inflammation, and in which the greater part of the phenomena will be based upon our physiological knowledge.

It is known that many authors, and in particular Dr. Barry, had attributed a very important part to atmospheric pressure in the phenomena of the circulation. The greater part of the sensations experienced by travellers who have ascended to great heights, were explained in this manner. To satisfy himself of the truth of these assertions, M. Poiseuille contrived an apparatus, which he calls the *pneumatic porte-object*, and by means of which he is able to examine, under the microscope, the capillary circulation in an animal, either by subjecting him to a pressure of two, three, and four atmospheres, or by reducing this pressure to two centimetres of mercury, that is to say, the thirty-eighth part of what it ordinarily is at the seaboard.* Thus the phenomena, which result from the rarefaction or extreme pressure of the atmosphere, depend upon the respiration, and not upon the capillary circulation, and the movements of inspiration and expiration are, no more than atmospheric pressure, indispensable causes of the circulation, but only accessary causes of the movement of the blood in the arteries and veins.

* He has satisfied himself that these differences of pressure have no influence upon the velocity of the capillary circulation.

